# Cartilage oligomeric matrix protein overexpression is an independent poor prognostic indicator in patients with intrahepatic cholangiocarcinoma

**DOI:** 10.1038/s41598-023-43006-z

**Published:** 2023-10-14

**Authors:** Khaa Hoo Ong, Yao-Yu Hsieh, Hong-Yue Lai, Ding-Ping Sun, Tzu-Ju Chen, Steven Kuan-Hua Huang, Yu-Feng Tian, Chia-Ling Chou, Yow-Ling Shiue, Hung-Chang Wu, Ti-Chun Chan, Hsin-Hwa Tsai, Chien-Feng Li, Po-An Su, Yu-Hsuan Kuo

**Affiliations:** 1 Division of Gastroenterology and General Surgery, Department of Surgery, Chi Mei Medical Center, Tainan, 710 Taiwan, ROC; 2 Department of Medical Technology, Chung Hwa University of Medical Technology, Tainan, 717 Taiwan, ROC; 3 Institute of Biomedical Sciences, National Sun Yat-Sen University, Kaohsiung, 804 Taiwan, ROC; 4 Division of Hematology and Oncology, Shuang Ho Hospital, Taipei Medical University, New Taipei City, 23561 Taiwan, ROC; 5 Division of Hematology and Oncology, Department of Internal Medicine, School of Medicine, College of Medicine, Taipei Medical University, Taipei, 11031 Taiwan, ROC; 6 Department of Pharmacology, School of Medicine, College of Medicine, China Medical University, Taichung, 404 Taiwan, ROC; 7 Department of Clinical Pathology, Chi Mei Medical Center, Tainan, 710 Taiwan, ROC; 8 Division of Urology, Department of Surgery, Chi Mei Medical Center, Tainan, 710 Taiwan, ROC; 9 Department of Medical Science Industries, College of Health Sciences, Chang Jung Christian University, Tainan, 711 Taiwan, ROC; 10 Division of Colon and Rectal Surgery, Department of Surgery, Chi Mei Medical Center, Tainan, 710 Taiwan, ROC; 11 Institute of Precision Medicine, National Sun Yat-Sen University, Kaohsiung, 804 Taiwan, ROC; 12 Division of Hematology and Oncology, Department of Internal Medicine, Chi-Mei Medical Center, Tainan, 71004 Taiwan, ROC; 13 College of Pharmacy and Science, Chia Nan University, Tainan, 71710 Taiwan, ROC; 14 Department of Medical Research, Chi Mei Medical Center, Tainan, 710 Taiwan, ROC; 15 National Institute of Cancer Research, National Health Research Institutes, Tainan, 704 Taiwan, ROC; 16 Department of Laboratory Medicine, China Medical University Hospital, Taichung, 404 Taiwan, ROC; 17 Trans-Omic Laboratory for Precision Medicine, Chi Mei Medical Center, Tainan, 710 Taiwan, ROC; 18 Department of Infectious Disease, Chi Mei Medical Center, No.901, Zhonghua Rd. Yongkang Dist, Tainan City, 71004 Taiwan, ROC

**Keywords:** Cancer, Biomarkers

## Abstract

Cartilage oligomeric matrix protein (COMP) interacts with various extracellular matrix proteins in tissues. Elevated COMP levels recently linked to worse overall survival in multiple cancer types. COMP's significance in intrahepatic cholangiocarcinoma (iCCA) remains uncertain. Here we report a retrospective study to explore COMP's impact on iCCA outcomes. We collected 182 patients' iCCA tumor tissues. COMP overexpression was associated with adverse factors like R1 resection (*p* = 0.008), advanced T stage (*p* < 0.001), large duct type (*p* = 0.004), and poorly differentiated histology (*p* = 0.002). COMP overexpression correlates with poorer DFS (HR, 3.651; *p* = 0.001), OS (HR, 1.827; *p* = 0.023), LRFS (HR, 4.077; *p* < 0.001), and MFS (HR, 3.718; *p* < 0.001). High COMP expression ties to worse overall survival (*p* = 0.0001), DSS (*p* < 0.0001), LRFS (*p* < 0.0001), and MFS (*p* < 0.0001). In conclusion, COMP overexpression links to poor prognosis and pathological features in iCCA, indicating its potential as a biomarker.

## Introduction

Previous studies have elucidated the pivotal role of cartilage oligomeric matrix protein (COMP), also known as thrombospondin-5 (TSP-5), in various cancers. COMP's presence in multiple cell types and its contributions to maintaining extracellular matrix (ECM) integrity have been established. Notably, its functions encompass stabilizing ECM protein connections and enhancing tissue mechanical strength. COMP levels are elevated in a variety of musculoskeletal disorders^1^. High COMP levels have also been associated to breast cancer, hepatocellular carcinoma, prostate cancer, and colon cancer. In these cancers, COMP overexpression was associated with increased tumor growth, cancer metastasis, cancer recurrence, and overall shorter survival^2–5^. Against this backdrop, the prognostic significance of COMP in intrahepatic cholangiocarcinoma (iCCA) remains unexplored.

After hepatocellular carcinoma (HCC), cholangiocarcinoma (CCA) is the second most prevalent primary hepatic malignancy, accounting for around 15% of all primary liver tumors and 3% of all gastrointestinal cancers^6,7^. CCA is a diverse collection of malignant cancers that arise in various parts of the biliary tree. CCAs are divided into three types: intrahepatic (iCCA), perihilar (pCCA), and distal CCA (dCCA), each with its own etiologies, risk factors, prognosis, and clinical and therapies. Together, iCCA and pCCA account for more than 90% of all CCAs in the world^8^. CCA is a rare cancer, but its incidence (0.3–6 per 100,000 people per year) and mortality (1–6 per 100,000 people per year, globally, excluding specific regions with incidence > 6 per 100,000 people, such as South Korea, China, and Thailand) have been rising in recent decades, posing a global health problem^9–11^. Over the past few decades, the reported age-standardized incidence for iCCA has been steadily increasing in most locations worldwide, whereas the age-standardized incidence for dCCA has been decreasing^12^. Curative surgery remained the standard treatment for early CCA. Despite the improvement in CCA pathogenesis, diagnosis, and treatments over the last decade, patient prognosis has remained unchanged, with 5-year survival rates of 7–20 percent and tumor recurrence rates were as high as 48–56% after resection remaining unsatisfactory^13–17^. Several clinical markers, including as T stage, lymph node metastases, and histological grade, can be used to identify high-risk patients^16^. However, to establish further treatment plans after curative surgery, genomics-based prognostic biomarkers were warranted.

Given the rising incidence and mortality of iCCA globally, coupled with the challenges in improving patient prognosis, an investigation into COMP's role in iCCA assumes importance. Therefore, the primary aim of this study is to assess COMP expression levels in iCCA patients and determine its potential as a prognostic marker. In summary, this study investigates the expression of COMP in intrahepatic cholangiocarcinoma (iCCA) and its implications for prognostic evaluation. By shedding light on COMP's potential as a prognostic marker, our work seeks to contribute valuable insights into enhancing the management and treatment outcomes of iCCA patients.

## Results

### Upregulation of COMP gene links to extracellular matrix structure in the CCA transcriptome

**Figure 1:**
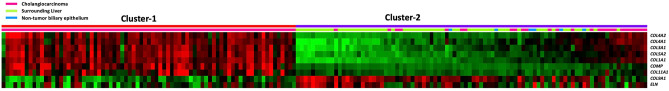
A published transcriptome dataset of intrahepatic cholangiocarcinoma (GSE26566) from GEO database showed gene expression associated with extracellular matrix structure constituent (GO:0005201). COMP is the most upregulated genes in cholangiocarcinoma compared to surrounding liver and normal biliary epithelium.

For data mining, a published CCA transcriptome dataset (GSE26566) was used, which includes 104 patients who had radical surgery. We found 9 probes that covered 9 transcripts related to structural elements of the extracellular matrix (GO:0,005,201) (Table 1 and Fig. 1). When compared to neighboring liver tissue, COMP gene (ILMN 1,677,636) was elevated by up to 2.3107-fold log ratios (*p* < 0.0001) in CCA, as shown in Table 1. When compared to the normal intrahepatic bile duct, COMP was also enhanced in CCA, with 2.4308-fold log ratios (*p* < 0.0001). Thus, COMP was chosen for further analysis.

**Table 1:** Summary of the alterations of gene associated with extracellular matrix structural constituent (GO:0005201) in cholangiocarcinoma (GSE26566).

Probe	CCA vs Non-tumor^#^		CCA vs Normal intrahepatic bile duct^&^		Gene symbol	Molecular funciton	Biological process
	Log ratio	*p*-value	Log ratio	*p*-value			
ILMN_1677636	2.3107	< 0.0001	2.4308	< 0.0001	*COMP*	Extracellular matrix structural constituent, calcium ion binding, protein binding	Skeletal development, cell adhesion
ILMN_1701308	2.1399	< 0.0001	3.0183	< 0.0001	*COL1A1*	Extracellular matrix structural constituent, structural constituent of bone	Skeletal development, phosphate transport, epidermis development
ILMN_1773079	1.468	0.0003	1.8736	< 0.0001	*COL3A1*	Extracellular matrix structural constituent	Phosphate transport, circulation
ILMN_1729117	1.2469	0.0008	1.8649	< 0.0001	*COL5A2*	Extracellular matrix structural constituent	Phosphate transport
ILMN_1789507	1.225	0.0026	1.278	< 0.0001	*COL11A1*	Extracellular matrix structural constituent, protein binding; bridging	Extracellular matrix organization and biogenesis, cartilage condensation, visual perception, cell–cell adhesion, phosphate transport
ILMN_1724994	0.8912	0.0024	1.4012	< 0.0001	*COL4A2*	Extracellular matrix structural constituent	Extracellular matrix organization and biogenesis, phosphate transport
ILMN_1653028	0.8428	0.0048	1.8243	< 0.0001	*COL4A1*	Extracellular matrix structural constituent	Phosphate transport
ILMN_1665374	-0.3279	0.0019	-0.4715	< 0.0001	*COL9A1*	Extracellular matrix structural constituent	Phosphate transport, cell adhesion
ILMN_1757506	-0.3085	0.0032	-0.1651	< 0.0001	*ELN*	Extracellular matrix structural constituent, extracellular matrix constituent conferring elasticity, GTP binding	Respiratory gaseous exchange, circulation, cell proliferation

^**#**^Comparing cholangiocarcinoma (CCA, n = 104) to surrounding liver (n = 59) and normal intrahepatic bile duct (n = 6); &, Comparing cholangiocarcinoma (CCA, n = 104) to normal intrahepatic bile duct (n = 6); * statistically significant.

### Clinicopathological features of the iCCA cohorts

Our study includes total 182 iCCA patients who had undergone radical surgery (Table 2). Males account for a slightly larger proportion (59.3%). Most patients (59%) were younger than 65 years old. 72 patients (40%) were chronic hepatitis B carrier, 29 (16%) patients were chronic hepatitis C carrier. Intrahepatic lithiasis was identified in 80 patients (44%). R0 resection was achieved in 163 patients (90%). 87 patients (48%) had T1 lesions, 61 patients (34%) had T2 lesions, while 34 patients (18%) had T3 lesions. Regarding histological findings, 105 patients (58%) had large duct type cholangiocarcinoma, while 77 patients (42%) had small duct type cholangiocarcinoma. 61 patients (34%) had well differentiated tumor, 66 patients (36%) had moderate differentiated tumor, and 55 patients (30%) had poorly differentiated tumor.

**Table 2:** Correlations between COMP expression and other important clinicopathological parameters in primary localized iCCA analyzed by Pearson's chi-square test.

Parameter	Category	Case no	COMP expression		*p*-value
			Low	High	
Gender	Male	108	53	55	0.763
	Female	74	38	36	
Age (years)	< 65	107	54	53	0.880
	≥ 65	75	37	38	
Hepatitis	Hepatitis B	72	34	38	0.578
	Hepatitis C	29	17	12	
	Non-B, non-C	81	40	41	
Intrahepatic lithiasis	Not identified	102	55	47	0.232
	Present	80	36	44	
Surgical margin	R0	163	87	76	0.008*
	R1	19	4	15	
Primary tumor (T)	T1	87	59	28	< 0.001*
	T2	61	25	36	
	T3	34	7	27	
Histological variants	Large duct type	105	43	62	0.004*
	Small duct type	77	48	29	
Histological grade	Well differentiated	61	38	23	0.020*
	Moderately differentiated	66	33	33	
	Poorly differentiated	55	20	25	

*Statistically significant.

### Correlations between COMP expression and pathological features in iCCA

**Figure 2:**
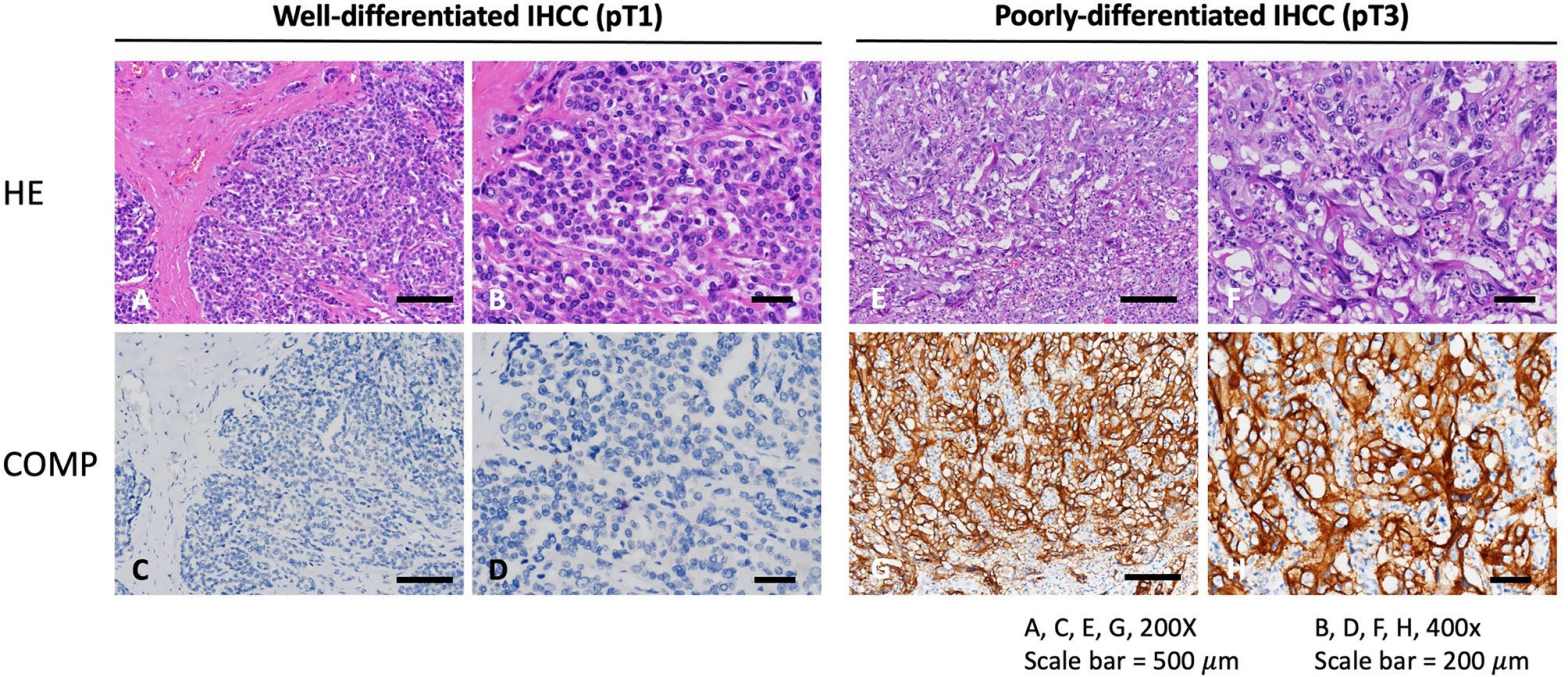
Immunohistochemistry staining showed lower COMP expression in well-differentiated and pT1 (**A**, **B**, **C**, **D**) iCCA compared to poorly-differentiated and pT3 stage (**E**, **F**, **G**, **H**) iCCA. (Magnification: **A**, **C**, **E**, **G**, 200X; **B**, **D**, **F**, **H**, 400X).

The association between COMP expression and various clinicopathological features was assessed using Pearson’s chi-square test. As determined using immunohistochemistry, poorly differentiated and higher T stage cholangiocarcinoma had greater COMP immunoreactivity than well differentiated and lower T stage cholangiocarcinoma (Fig. 2). Table 2 summarized the relationships between COMP expression levels and clinicopathological parameters in iCCA cases. COMP overexpression was associated with R1 resection (*p* = 0.008), advanced T stage (*p* < 0.001), large duct type (*p* = 0.004) and poor differentiated histology (*p* = 0.002).

### Prognostic significance of COMP expression

The predictive effects of COMP expression on cancer metastasis and patient survival in iCCA were assessed using univariate and multivariate analyses. (Table 3). In univariate analysis, female gender, R1 resection, advanced T stage and COMP overexpression were significantly associated with worse disease-specific survival (DSS) and overall survival (OS) (Table 3). To further evaluate the recurrence pattern, R1 resection, advanced T stage, large duct type CCA, well differentiated CCA and high COMP expression were revealed to be significant predictive factors for shorter local recurrence-free survival (LRFS). While R1 resection, advanced T stage and high COMP expression were significantly linked to worse metastases-free survival (MFS) (Table 4). Multivariate Cox Regression analysis revealed that R1 resection, advanced T stage and high COMP expression were significant indicators for worse DSS and OS (Table 3). Only R1 section and high COMP expression were associated with shorter LRFS, while R1 resection, advanced T stage and high COMP expression were significantly associated with poor MFS (Table 4). COMP overexpression was found to be substantially linked with poor DFS (hazard ratio [HR], 3.651; 95% confidence interval [CI], 1.675–7.958; *p* = 0.001), poor OS (HR, 1.827; 95% CI, 1.087–3.070; *p* = 0.023), poor LRFS (HR, 4.077; 95% CI, 2.350–7.072; *p* < 0.001), and poor MFS (HR, 3.718; 95% CI, 2.026–6.823; *p* < 0.001) in multivariate analysis (Tables 3 and 4).

**Table 3:** Univariate log-rank and multivariate analyses for overall and disease-specific survivals in primary localized iCCA.

Parameter	Category	Case No	Overall Survival					Disease-specific Survival				
			Univariate analysis		Multivariate analysis			Univariate analysis		Multivariate analysis		
			No. of event	*p*-value	R.R	95% C.I	*p*-value	No. of event	*p*-value	R.R	95% C.I	*p*-value
Gender	Male	108	50	0.0254*	1	–	0.051	9	0.0072*	1	–	0.016*
	Female	74	21		1.664	0.997–2.777	–	32		2.158	1.187–5.255	–
Age (years)	< 65	107	37	0.2626	–	–	–	28	0.2125	–	–	–
	≥ 65	75	34		–	–	–	13		–	–	–
Hepatitis	Hepatitis B	72	32	0.2379	–	–	–	16	0.4561	–	–	–
	Hepatitis C	29	8		–	–	–	19		–	–	–
	Non-B, non-C	81	31		–	–	–	6		–	–	–
Intrahepatic lithiasis	Not identified	102	36	0.2831	–	–	–	19	0.1613	–	–	–
	Present	80	35		–	–	–	22		–	–	–
Surgical margin	R0	163	59	< 0.0001*	1	–	0.005*	31	< 0.0001*	1	–	0.002*
	R1	19	12		2.563	1.294–5.073		10		3.554	1.609–7.845	
Primary tumor (T)	T1	87	25	0.0001*	1	–	0.015*	9	< 0.0001*	1	–	0.009*
	T2	61	27		1.518	0.862–2.673	–	19		2.481	1.099–5.604	–
	T3	34	19		2.236	1.160–4.311	–	13		3.256	1.317–8.051	–
Histological variants	Large duct type	105	43	0.4281	–	–	–	27	0.1984	–	–	–
	Small duct type	77	28		–	–	–	14		–	–	–
Histological grade (differentiation)	Well	61	20	0.1663	–	–	–	12	0.3881	–	–	–
	Moderately	66	28		–	–	–	16		–	–	–
	Poorly	55	23		–	–	–	13		–	–	–
COMP Exp	Low expression	91	27	0.0001	1	–	0.023*–	9	< 0.0001*	1	–	0.001*–
	High expression	91	44		1.827	1.087–3.070	–	32		3.651	1.675–7.958	–

*Statistically significant.

**Table 4:** Univariate log-rank and multivariate analyses for local recurrence-free and metastasis-free survivals in primary localized iCCA.

Parameter	Category	Case No	Local recurrence-free survival					Metastasis-free survival				
			Univariate analysis		Multivariate analysis			Univariate analysis		Multivariate analysis		
			No. of event	*p*-value	R.R	95% C.I	*p*-value	No. of event	*p*-value	R.R	95% C.I	*p*-value
Gender	Male	108	54	0.2170	–	–	–	21	0.1008	–	–	–
	Female	74	31		–	–	–	44		–	–	–
Age (years)	< 65	107	55	0.2993	–	–	–	42	0.2936	–	–	–
	≥ 65	75	30		–	–	–	23		–	–	–
Hepatitis	Hepatitis B	72	33	0.7333	–	–	–	26	0.8762	–	–	–
	Hepatitis C	29	13		–	–	–	11		–	–	–
	Non-B, non-C	81	39		–	–	–	28		–	–	–
Intrahepatic lithiasis	Not identified	102	41	0.0551	–	–	–	31	0.1000	–	–	–
	Present	80	44		–	–	–	34		–	–	–
Surgical margin	R0	163	71	< 0.0001*	1	–	0.001*	54	< 0.0001*	1		0.038*
	R1	19	14		2.886	1.506–5.530		11		2.122	1.042–4.320	
Primary tumor (T)	T1	87	28	< 0.0001*	1	–	0.022*	21	< 0.0001*	1	–	0.048*
	T2	61	32		1.283	0.739–2.228		26		1.563	0.856–2.854	
	T3	34	25		1.823	0.971–3.423		18		1.981	0.992–3.955	
Histological variants	Large duct type	105	58	0.0085*	1	–	0.570	43	0.0759	–	–	–
	Small duct type	77	27		0.870	0.537–1.408		22		–	–	–
Histological grade (Differentiation)	Well	61	28	0.0299*	1	–	0.765	22	0.1794	–	–	–
	Moderately	66	27		0.914	0.534–1.564		22		–	–	–
	Poorly	55	30		0.764	0.435–2.1.342		21		–	–	–
COMP Exp	Low expression	91	21	< 0.0001*	1	–	< 0.001	16	< 0.0001*	1	–	< 0.001*
	High expression	91	64		4.077	2.350–7.072		49		3.718	2.026–6.823	

*Statistically significant.

### Survival analysis in iCCA

**Figure 3:**
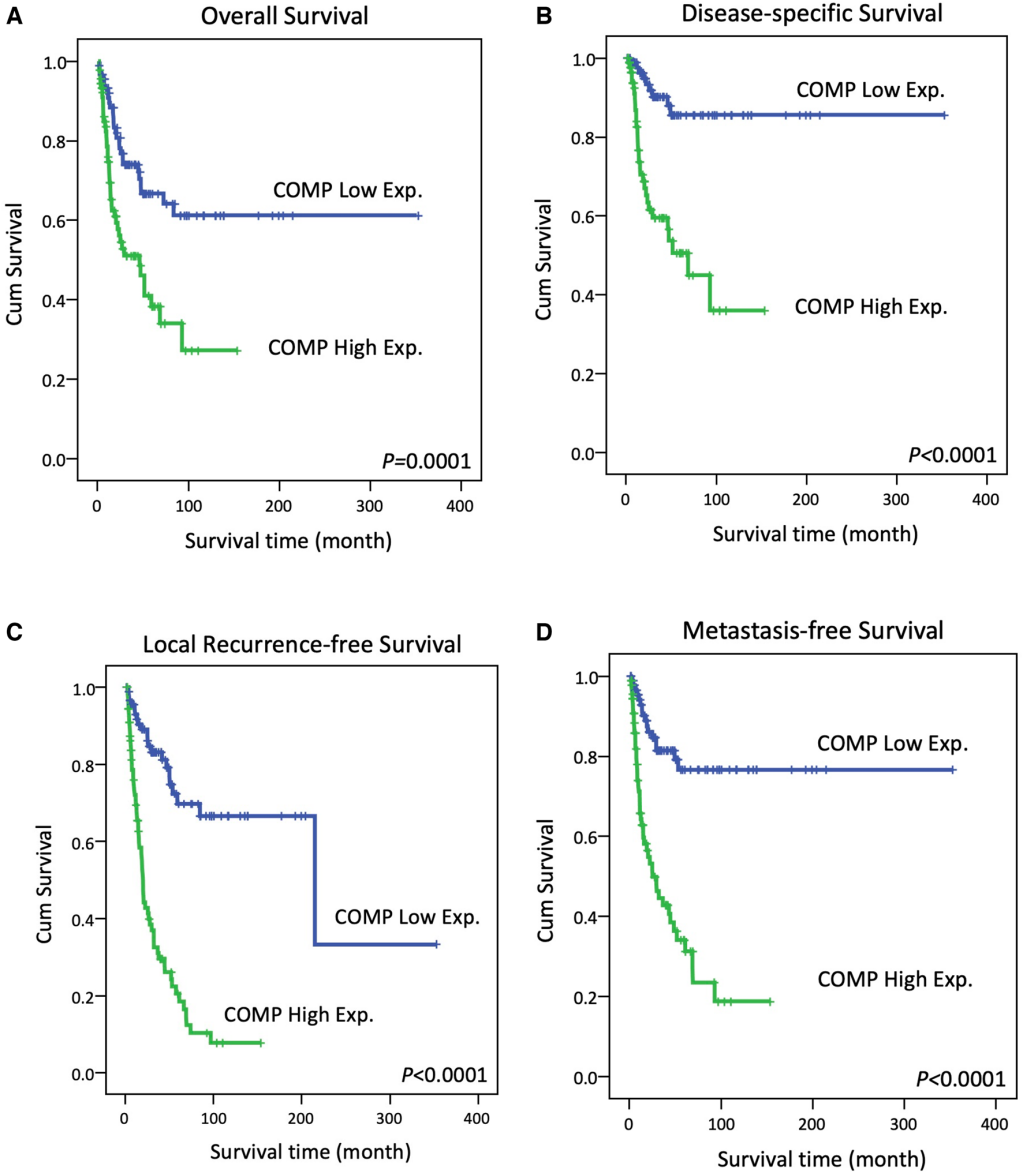
Kaplan–Meier analysis showed COMP overexpression associated with (**A**) worse overall survival (**B**) worse disease-specific survival (**C**) worse local recurrence free survival (**D**) worse metastasis free survival in iCCA patient.

Kaplan–Meier analysis showed COMP overexpression was correlated with poorer overall survival (Fig. 3A; *p* = 0.0001), DSS (Fig. 3B; *p* < 0.0001), LRFS (Fig. 3C; p < 0.0001) and MFS (Fig. 3D; p < 0.0001) in iCCA patients.

### COMP gene function prediction

**Figure 4:**
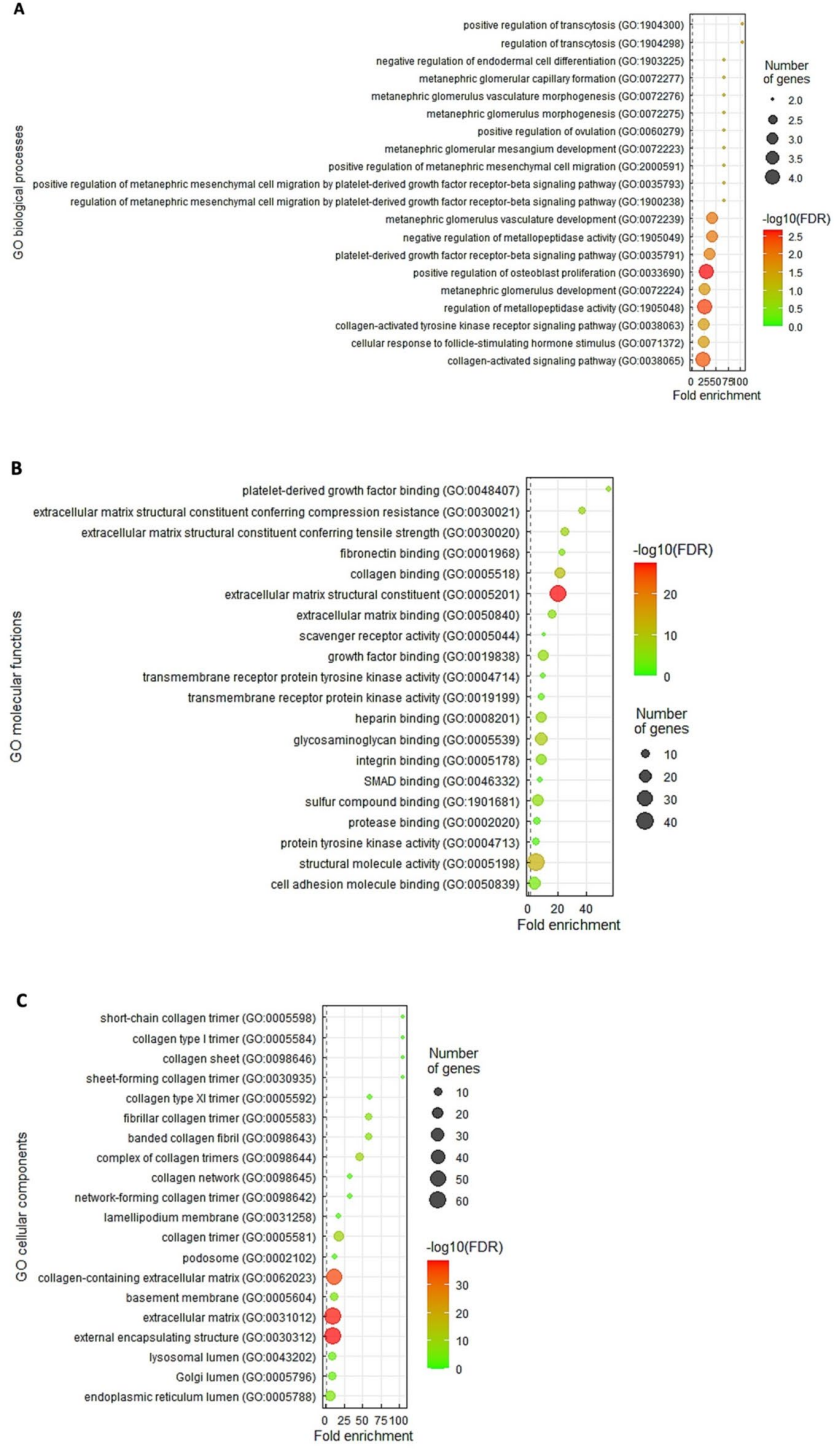
The outstanding GO terms enriched in *COMP* upregulation. Employing the GO classification system based on (**A**) biological processes, (**B**) molecular functions, and (**C**) cellular components, the top 20 GO terms presenting positive correlations with *COMP* were displayed and ordered by fold enrichment.

To recognize the potential functions of *COMP* in IHCC, we downloaded the top two hundred differentially expressed transcripts showing positive connections (Supplementary Table 1) or negative connections (Supplementary Table 2) with *COMP* from the cholangiocarcinoma dataset (TCGA, *n* = 51). Later, employing the Gene Ontology (GO) classification system, these transcripts were utilized to prognosticate the functions of *COMP*. The results revealed that, in respect to biological processes (Fig. 4A), the most significant term correlated with *COMP* upregulation was positive regulation of transcytosis (GO: 1,904,300, fold enrichment: > 100), and the platelet-activating factor receptor (*PTAFR*) gene was identified. In view of molecular functions (Fig. 4B), the most remarkable term correlated with *COMP* upregulation was platelet-derived growth factor binding (GO: 0,048,407, fold enrichment: 55.32) that includes the platelet-derived growth factor subunit B (*PDGFB*), platelet-derived growth factor receptor beta (*PDGFRB*), collagen type I alpha 1 chain (*COL1A1*), *COL1A2*, *COL3A1*, and *COL5A1* genes. Furthermore, with regard to cellular components (Fig. 4C), the most important terms correlated with *COMP* upregulation were short-chain collagen trimer (GO: 0,005,598, fold enrichment: > 100), collagen type I trimer (GO: 0,005,584, fold enrichment: > 100), and collagen sheet (GO: 0,098,646, fold enrichment: > 100).

## Discussion

In contrast to HCCs, mass-forming iCCAs are characterized by a significant desmoplastic and hypovascularized tumor stroma, which is frequently the predominant histological characteristic of the tumor^18^. Several research have studied not only the morphological differences, but also the molecular distinctions between iCCA, pCCA, and dCCA^19–21^. Nakamura et al. demonstrated a variation in tumor anatomical location, indicating IDH, EPHA2 and BAP1 mutations and FGFR2 fusions in iCCA, whereas extrahepatic tumors preferentially show PRKACA and PRKACB fusions as well as mutations in ELF3 and ARID1B. Based on these findings, cholangiocarcinoma in different anatomical locations should be treated and examined differently^20^. Furthermore, cholangiocarcinoma's highly desmoplastic nature, substantial support from a rich tumor microenvironment, and significant genetic heterogeneity all contribute to its treatment resistance^22^.

COMP is a large pentameric cartilage protein and is found in articular cartilage, ligaments, and tendons. The protein can also be found in the skin, breast tissues, and liver tissues^3,23,24^. Although the function of COMP glycoprotein in a number of connective tissue illnesses has been well documented^25^, its function in carcinogenesis is still unknown. Evidence from recent studies has shown that COMP overexpression is associated with a poor prognosis and metastases in breast, prostate, thyroid, colon, and hepatocellular carcinoma^2,3,5,26–28^. In our previous study, we found COMP overexpression associated with poor survival and tumor invasiveness in urothelial carcinoma^29^.

As the extracellular matrix degrades with continued liver damage and fibrotic scar tissue builds up, the liver undergoes remodeling, which eventually results in fibrosis, cirrhosis, and ultimately hepatocellular carcinoma (HCC). Norman et el. demonstrated serum COMP could be an early marker of fibrosis, and that increased serum COMP levels could reflect the degree of cartilage breakdown during liver destruction and re- modeling, and also associated with HCC development^2^. Liver cirrhosis and viral hepatitis C and B have been recognized as risk factors for cholangiocarcinoma, especially intrahepatic disease^30–33^. The following associations were found in a meta-analysis^34^ of several case–control studies on the risk factors for intrahepatic cholangiocarcinoma: cirrhosis had a combined odds ratio (OR) of 22·92 (95% CI 18·24–28·79), hepatitis C of 4·84 (2·41–9·71), and hepatitis B of 5·10 (2·91–8·95). Based on this, we think that the COMP may also affect the prognosis of iCAA due to the shared etiology of HCC and iCCA. Moreover, cholangiocarcinoma is usually characterized by a prominent desmoplastic and hypovascularized stroma. There is now increasing evidence to suggest that the desmoplastic reaction, marked by a dramatic accumulation of α-smooth muscle actin positive cancer-associated fibroblasts (α-SMA + CAFs) with increased production of extracellular matrix proteins^18^. Based on these, we use the transcriptome dataset to assess the genes related to extracellular matrix structure constituent (GO:0005201). When compared to neighboring liver tissue, COMP gene was significantly elevated in CCA among these genes. Thus, COMP was chosen as a candidate for further survival analysis. To our knowledge, this is the first study to demonstrate COMP overexpression associated with poor survival in iCCA.

The Notch, WNT, and transforming growth factor (TGF) signaling pathways, among others, are highly active in iCCA. It is known that the Notch pathway has a role in biliary repair, growth, fibrosis, and stem cell niche maintenance. NOTCH3 overexpression was linked to the development and progression of iCCA, promoting cell survival through PI3K-AKT signaling^12,35,36^. Interestingly, Konstantinos et al. demonstrated that COMP regulates the cancer stem cell population through increasing the interaction between Notch3 and Jagged1, leading to increased activation of Notch3 signaling^37^.

In the other way, the epithelial to mesenchymal transition (EMT) is a critical biological step in the migration and invasion of malignant tumor cells^38^. It gives epithelial cancer cells the ability to develop mesenchymal characteristics with invasive capabilities, which promote colonization of metastatic sites^39^. The TGF-dependent pathway, whose signature has been discovered in iCCA stroma, is the prototypical inducer of EMT. In CCA, TGF either directly induces EMT or collaborates with other key EMT inducer pathways like EGFR^40–42^. Notably, transcription factors (EMT-TFs) that control the expression of epithelial and mesenchymal genes, including as the SNAIL, ZEB, and TWIST families, orchestrate EMT. Regardless of anatomical location, CCAs express EMT-TFs, which are linked to a poor prognosis^12,43,44^. Interestingly, COMP can stimulate tumor EMT, although the mechanism is unknown^27,45^. COMP glycoprotein has been demonstrated to be co-expressed with many EMT genes, and a clear association between high COMP expression and poor colon cancer survival has also been reported^45,46^. The evidences to support the link of NOTCH3 pathway, EMT pathway between COMP and iCCA are ambiguous initially but gradually apparent. However, the exact mechanism by which COMP acts as a poor prognostic factor in iCCA remains unclear and need further exploration.

Recently, microRNAs (miRs) and extracellular vesicles (EVs) have gained attention as promising non-invasive biomarkers for diagnosis and prognosis in cholangiocarcinoma^47^. MiRs can be secreted extracellularly or encapsulated in EVs, thereby taking part in intercellular communication^48^. Transcytosis, a type of transcellular mechanism for crossing of EVs through the interior of a cell, has also been implicated in various solid tumors^49^. Interestingly, the results of our bioinformatic analysis revealed that, in terms of biological processes, the most significant term correlated with *COMP* upregulation was positive regulation of transcytosis (Fig. 4A) that contains the *PTAFR* gene. Activation of the platelet-activating factor (PAF)/PTAFR pathways has also been suggested to cause hepatocellular carcinoma cell migration and invasion^50^. However, whether COMP may promote IHCC progression through transcytosis and PTAFR needs further investigation. Moreover, in terms of molecular functions, the most remarkable term correlated with *COMP* upregulation was platelet-derived growth factor binding (Fig. 4B) that includes the *PDGFB*, *PDGFRB*, *COL1A1*, *COL1A2*, *COL3A1*, and *COL5A1* genes. The critical role of the PDGFB/PDGFRB axis in angiogenesis has been demonstrated in tumors^51^. PDGFD can also bind PDGFRB and activate cancer-associated fibroblasts (CAFs) that play crucial roles in modulating cholangiocarcinoma development^52^. The *COL1A1*, *COL1A2*, *COL3A1*, and *COL5A1* genes are also identified as CAF signature in cholangiocarcinoma^53,54^. Accordingly, the associations among the expression levels of COMP, platelet-derived growth factors, and CAF-derived collagen in IHCC development are quite interesting and deserve further examination.

Our research has certain limitations. Firstly, it is a retrospective study conducted at a single institution and lack of experimental validation. Secondly, the exact molecular mechanism underlying disease progression and adverse outcomes in COMP-overexpressing IHCC remains unclear. Thirdly, there is currently no standardized immunostaining and scoring scheme for assessing COMP expression. Due to the lack of agreed staining standards, it is difficult to reach a consensus in this type of research. Fourthly, The GEO transcriptome dataset we use is from cholangiocarcinoma patients rather than iCCA patients, which may also cause some limitations in this study. Lastly, to validate our findings, prospective multicenter studies are required.

In conclusion, COMP overexpression was associated with worsening clinical-pathological characteristics. COMP is also related with poorer survival in iCCA, supporting its function as a biomarker for iCCA prognosis. This is the first study to our knowledge that clarifies the role of COMP in iCCA. To completely comprehend the mechanism and apply these findings to clinical practice, additional research is required.

## Methods

### Data mining of the gene expression omnibus (GEO) dataset

A transcriptome dataset (GSE26566) containing 104 iCCA patients who received curative surgery was obtained from the NCBI Gene Expression Omnibus (GEO) database. Without pre-selection, all probe sets were used. The raw data was then transferred into the Nexus Expression 3 software, which was used to calculate gene expression levels. Comparative analyses were conducted to detect the significantly differently expressed genes connected to extracellular matrix structural constituents (GO:0,005,201) by comparing CCA part vs. normal surrounding liver and CCA part vs. normal intrahepatic duct. After analysis, differentially regulated genes in CCA part vs. normal surrounding liver and CCA part vs. normal intrahepatic duct were discovered (P < 0.0001 and log ratio > 2) (Fig. 1 and Table 1).

### Study population

Between 1990 and 2010, 182 patients with iCCA who received curative surgery were enrolled at Chi Mei Medical Center. The presence of lymph node involvement or distant metastasis was ruled out to guarantee curability. Only individuals with T1-3N0M0 disease were included. Two pathologists investigated tumor samples to rule out the possibility of other malignancies arising from the biliary system. In this study, we used anonymous patient sample information from biobank as approved by IRB. As a rule, inform consent has been sign by every patient before their sample/information collected into biobank. The study had been approved by the Institutional Review Board (IRB) of Chi Mei Medical Center with the approval number of 09,912,003. All research was performed in accordance with relevant guidelines/regulations. We gathered patients' retrospective demographic and clinical data, including pathological characteristics, oncological survival follow-up, and cause of mortality. Patients with acute blood diseases, abnormal bone marrow, concurrent cancer, or insufficient clinical data were excluded from the research. The tumor stage was assessed using the eighth edition of American Joint Committee on Cancer (AJCC) Tumor, Node, Metastasis (TNM) system developed in 2017. This study followed the REMARK (Reporting Recommendations for Tumor Marker Prognostic Studies) guidelines.

### Immunohistochemistry and scoring

Specimens were prepared according to protocol. The primary COMP antibody was incubated for 1 h on the sections (Clone: EPR22857-38, Abcam). Then the primary antibody was diluted 1:200 in TBS with 1% BSA. Antibodies were then detected using a DAKO ChemMate EnVision Kit (K5001, Carpinteria, CA, USA). Cell blocks from cell lines known to express COMP were used as positive controls. Sections were processed without the primary anti-COMP antibody as negative controls. Two pathologists used the following equation to calculate the H-score to estimate COMP immunoreactivity: H-score = SPi (i + 1), where Pi is the percentage of stained tumor cells in various intensities ranging from 0 to 100%, and I is the degree of staining (0 to 3 +). If there were any scoring disagreements, the two pathologists assessed the slides at the same time and agreed on an H-score. The immunostaining was categorized into low and high expression levels based on the median H-score as previously mentioned^55^.

### Functional gene annotation

To recognize the potential functions of *COMP* in intrahepatic cholangiocarcinoma, we appraised the relations between the mRNA expression levels of *COMP* and its coexpressed genes from the cholangiocarcinoma dataset (Firehose Legacy,* n* = 51) in The Cancer Genome Atlas (TCGA) database. Later, the top 200 differentially expressed transcripts showing positive connections or negative connections with *COMP* were downloaded. These transcripts were then annotated by the Gene Ontology (GO) classification system and ranked by fold enrichment.

### Statistical analysis

The connection between COMP expression and other clinicopathological characteristics was assessed using Pearson's chi-square test. We measured two outcomes: metastasis-free survival (MFS) and disease-specific survival (DSS). Using univariate and multivariate analysis, relevant COMP expression and clinicopathological variables were discovered as predictors of DSS (measured from curative surgery to the time of cancer mortality) and MFS (measured from curative surgery to the first metastasis). The survival curves were created using the Kaplan–Meier method and a log-rank test. All relevant parameters from the univariate analysis were integrated into the multivariate Cox proportional hazards model to find the independent variables. IBM's SPSS Statistics V.17.0 software (Armonk, NY, USA) was utilized for statistical analysis. The statistical significance criterion was chosen at P < 0.05.

### Supplementary Information

Supplementary Information.

## Data Availability

The transcriptome dataset (GSE26566) analyzed in the current study is available in a published archive from the Gene Expression Omnibus (GEO) database (National Center for Biotechnology Information, Bethesda, MD, USA). https://www.ncbi.nlm.nih.gov/geo/. The evaluation of *COMP* coexpressed genes was available in The Cancer Genome Atlas (TCGA) database. (National Cancer Institute and National Human Genome Research Institute, USA). https://www.cancer.gov/ccg/research/genome-sequencing/tcga.
