# Supplementary material for: Cartilage oligomeric matrix protein overexpression is an independent poor prognostic indicator in patients with intrahepatic cholangiocarcinoma
Source: Sci Rep. 2023 Oct 14;13:17444. doi: 10.1038/s41598-023-43006-z (PMC10576746; doi:10.1038/s41598-023-43006-z)
Supplement: Supplementary file 1 — Supplementary Information. [file 41598_2023_43006_MOESM1_ESM.docx]

**Supplementary Table 1. The top 200 genes positively correlated with COMP.**

| **Correlated Gene** | **Cytoband** | **Spearman's Correlation** | **p-Value** | **q-Value** |
| --- | --- | --- | --- | --- |
| **COL10A1** | 6q22.1 | 0.86 | 1.83E-11 | **1.91E-07** |
| **ANTXR1** | 2p13.3 | 0.859 | 2.11E-11 | **1.91E-07** |
| **LTBP2** | 14q24.3 | 0.855 | 3.15E-11 | **1.91E-07** |
| **INHBA** | 7p14.1 | 0.853 | 3.93E-11 | **1.91E-07** |
| **COL8A1** | 3q12.1 | 0.846 | 7.94E-11 | **3.09E-07** |
| **THBS2** | 6q27 | 0.843 | 1.09E-10 | **3.52E-07** |
| **AEBP1** | 7p13 | 0.819 | 1.00E-09 | **2.78E-06** |
| **MRC2** | 17q23.2 | 0.801 | 4.34E-09 | **1.06E-05** |
| **FAP** | 2q24.2 | 0.792 | 9.06E-09 | **1.96E-05** |
| **CDH11** | 16q21 | 0.786 | 1.34E-08 | **2.60E-05** |
| **ANOS1** | Xp22.31 | 0.782 | 1.79E-08 | **3.16E-05** |
| **LUM** | 12q21.33 | 0.773 | 3.24E-08 | **5.25E-05** |
| **CTHRC1** | 8q22.3 | 0.771 | 3.72E-08 | **5.25E-05** |
| **ITGBL1** | 13q33.1 | 0.771 | 3.78E-08 | **5.25E-05** |
| **COL11A1** | 1p21.1 | 0.768 | 4.69E-08 | **5.72E-05** |
| **PRELP** | 1q32.1 | 0.768 | 4.70E-08 | **5.72E-05** |
| **COL3A1** | 2q32.2 | 0.762 | 6.86E-08 | **7.85E-05** |
| **PODNL1** | 19p13.12 | 0.76 | 7.56E-08 | **7.86E-05** |
| **PGR** | 11q22.1 | 0.76 | 7.68E-08 | **7.86E-05** |
| **SRP72** | 4q12 | 0.757 | 9.01E-08 | **8.50E-05** |
| **CORIN** | 4p12 | 0.756 | 9.44E-08 | **8.50E-05** |
| **ACTBL2** | 5q11.2 | 0.756 | 9.77E-08 | **8.50E-05** |
| **FNDC1** | 6q25.3 | 0.755 | 1.01E-07 | **8.50E-05** |
| **CCN2** | 6q23.2 | 0.752 | 1.27E-07 | **9.88E-05** |
| **ANGPTL2** | 9q33.3 | 0.752 | 1.27E-07 | **9.88E-05** |
| **SFRP4** | 7p14.1 | 0.751 | 1.33E-07 | **9.95E-05** |
| **TMEM200A** | 6q23.1 | 0.749 | 1.46E-07 | **1.05E-04** |
| **MXRA8** | 1p36.33 | 0.746 | 1.80E-07 | **1.25E-04** |
| **CBLN4** | 20q13.2 | 0.744 | 2.02E-07 | **1.35E-04** |
| **NTM** | 11q25 | 0.741 | 2.32E-07 | **1.50E-04** |
| **C1QTNF7** | 4p15.32 | 0.737 | 2.97E-07 | **1.87E-04** |
| **MXRA5** | Xp22.33 | 0.736 | 3.10E-07 | **1.89E-04** |
| **WNT2** | 7q31.2 | 0.736 | 3.20E-07 | **1.89E-04** |
| **ISLR** | 15q24.1 | 0.735 | 3.34E-07 | **1.91E-04** |
| **F2R** | 5q13.3 | 0.728 | 4.88E-07 | **2.71E-04** |
| **CYTH3** | 7p22.1 | 0.727 | 5.02E-07 | **2.71E-04** |
| **LDLR** | 19p13.2 | 0.725 | 5.84E-07 | **3.07E-04** |
| **CCN4** | 8q24.22 | 0.723 | 6.25E-07 | **3.20E-04** |
| **COL5A1** | 9q34.3 | 0.723 | 6.42E-07 | **3.20E-04** |
| **COL5A2** | 2q32.2 | 0.72 | 7.35E-07 | **3.57E-04** |
| **PDGFRB** | 5q32 | 0.72 | 7.55E-07 | **3.58E-04** |
| **DCN** | 12q21.33 | 0.717 | 8.51E-07 | **3.94E-04** |
| **GLI2** | 2q14.2 | 0.716 | 9.33E-07 | **4.22E-04** |
| **PLAT** | 8p11.21 | 0.714 | 1.02E-06 | **4.38E-04** |
| **SCARF2** | 22q11.21 | 0.714 | 1.04E-06 | **4.38E-04** |
| **HEPH** | Xq12 | 0.714 | 1.04E-06 | **4.38E-04** |
| **PDZRN3** | 3p13 | 0.711 | 1.15E-06 | **4.66E-04** |
| **TNS1** | 2q35 | 0.71 | 1.23E-06 | **4.87E-04** |
| **LAMA2** | 6q22.33 | 0.71 | 1.26E-06 | **4.89E-04** |
| **FUT11** | 10q22.2 | 0.709 | 1.31E-06 | **4.91E-04** |
| **ZMIZ1** | 10q22.3 | 0.709 | 1.32E-06 | **4.91E-04** |
| **TNFSF18** | 1q25.1 | 0.708 | 1.34E-06 | **4.91E-04** |
| **ARHGEF17** | 11q13.4 | 0.701 | 1.93E-06 | **6.96E-04** |
| **ISM1** | 20p12.1 | 0.699 | 2.11E-06 | **7.45E-04** |
| **GLT8D2** | 12q23.3 | 0.698 | 2.19E-06 | **7.59E-04** |
| **ADAMTS2** | 5q35.3 | 0.696 | 2.41E-06 | **8.22E-04** |
| **AXL** | 19q13.2 | 0.696 | 2.47E-06 | **8.28E-04** |
| **CCN1** | 1p22.3 | 0.694 | 2.72E-06 | **8.96E-04** |
| **ITGA11** | 15q23 | 0.691 | 3.14E-06 | **1.00E-03** |
| **COL6A3** | 2q37.3 | 0.69 | 3.29E-06 | **1.02E-03** |
| **TPM4** | 19p13.12-p13.11 | 0.69 | 3.29E-06 | **1.02E-03** |
| **LOX** | 5q23.1 | 0.686 | 3.84E-06 | **1.17E-03** |
| **KLF7** | 2q33.3 | 0.685 | 4.02E-06 | **1.20E-03** |
| **BGN** | Xq28 | 0.685 | 4.11E-06 | **1.21E-03** |
| **VCAN** | 5q14.2-q14.3 | 0.677 | 5.80E-06 | **1.68E-03** |
| **GXYLT2** | 3p13 | 0.677 | 5.89E-06 | **1.68E-03** |
| **NAV3** | 12q21.2 | 0.675 | 6.20E-06 | **1.75E-03** |
| **ANXA5** | 4q27 | 0.674 | 6.49E-06 | **1.80E-03** |
| **ITGAV** | 2q32.1 | 0.674 | 6.56E-06 | **1.80E-03** |
| **SULF1** | 8q13.2-q13.3 | 0.673 | 6.94E-06 | **1.85E-03** |
| **EDIL3** | 5q14.3 | 0.673 | 6.96E-06 | **1.85E-03** |
| **TSHZ3** | 19q12 | 0.672 | 7.33E-06 | **1.93E-03** |
| **G3BP2** | 4q21.1 | 0.671 | 7.49E-06 | **1.94E-03** |
| **IGFL2** | 19q13.32 | 0.671 | 7.61E-06 | **1.95E-03** |
| **APBB2** | 4p14-p13 | 0.667 | 8.83E-06 | **2.23E-03** |
| **MRVI1** | 11p15.4 | 0.666 | 9.22E-06 | **2.30E-03** |
| **COL1A2** | 7q21.3 | 0.662 | 1.08E-05 | **2.66E-03** |
| **HSPB7** | 1p36.13 | 0.662 | 1.10E-05 | **2.66E-03** |
| **MYADM** | 19q13.42 | 0.661 | 1.13E-05 | **2.72E-03** |
| **SGIP1** | 1p31.3 | 0.66 | 1.21E-05 | **2.86E-03** |
| **PODN** | 1p32.3 | 0.659 | 1.26E-05 | **2.94E-03** |
| **UNC5C** | 4q22.3 | 0.658 | 1.27E-05 | **2.94E-03** |
| **SSC5D** | 19q13.42 | 0.657 | 1.37E-05 | **3.13E-03** |
| **EGR2** | 10q21.3 | 0.656 | 1.43E-05 | **3.22E-03** |
| **ANO1** | 11q13.3 | 0.654 | 1.53E-05 | **3.43E-03** |
| **SRFBP1** | 5q23.1 | 0.653 | 1.57E-05 | **3.46E-03** |
| **HECW1** | 7p14.1-p13 | 0.651 | 1.71E-05 | **3.71E-03** |
| **PLPP4** | 10q26.12 | 0.651 | 1.72E-05 | **3.71E-03** |
| **KERA** | 12q21.33 | 0.65 | 1.78E-05 | **3.81E-03** |
| **SGCD** | 5q33.2-q33.3 | 0.649 | 1.83E-05 | **3.86E-03** |
| **KIF26B** | 1q44 | 0.649 | 1.86E-05 | **3.89E-03** |
| **ADAMTS12** | 5p13.3-p13.2 | 0.648 | 1.92E-05 | **3.97E-03** |
| **EDNRA** | 4q31.22-q31.23 | 0.648 | 1.94E-05 | **3.97E-03** |
| **DDR2** | 1q23.3 | 0.646 | 2.10E-05 | **4.26E-03** |
| **MAMDC2** | 9q21.12 | 0.646 | 2.12E-05 | **4.26E-03** |
| **CCL11** | 17q12 | 0.645 | 2.19E-05 | **4.30E-03** |
| **TANC2** | 17q23.2-q23.3 | 0.645 | 2.19E-05 | **4.30E-03** |
| **SERTAD4-AS1** | 1q32.2 | 0.644 | 2.30E-05 | **4.39E-03** |
| **SVIL** | 10p11.23 | 0.644 | 2.30E-05 | **4.39E-03** |
| **CPA4** | 7q32.2 | 0.641 | 2.51E-05 | **4.74E-03** |
| **MAP1A** | 15q15.3 | 0.641 | 2.57E-05 | **4.75E-03** |
| **TCF21** | 6q23.2 | 0.641 | 2.57E-05 | **4.75E-03** |
| **CPED1** | 7q31.31 | 0.64 | 2.59E-05 | **4.75E-03** |
| **LRP1** | 12q13.3 | 0.64 | 2.64E-05 | **4.80E-03** |
| **GNRHR** | 4q13.2 | 0.639 | 2.71E-05 | **4.82E-03** |
| **RFLNA** | 12q24.31 | 0.639 | 2.72E-05 | **4.82E-03** |
| **P4HA3** | 11q13.4 | 0.639 | 2.75E-05 | **4.82E-03** |
| **PARD3B** | 2q33.3 | 0.639 | 2.75E-05 | **4.82E-03** |
| **BBS12** | 4q27 | 0.639 | 2.78E-05 | **4.82E-03** |
| **PCDH10** | 4q28.3 | 0.636 | 3.08E-05 | **5.27E-03** |
| **HMCN1** | 1q25.3-q31.1 | 0.636 | 3.09E-05 | **5.27E-03** |
| **DKK3** | 11p15.3 | 0.636 | 3.12E-05 | **5.28E-03** |
| **ASPN** | 9q22.31 | 0.634 | 3.31E-05 | **5.54E-03** |
| **COL4A2** | 13q34 | 0.633 | 3.37E-05 | **5.60E-03** |
| **SOX6** | 11p15.2 | 0.632 | 3.54E-05 | **5.83E-03** |
| **COL1A1** | 17q21.33 | 0.632 | 3.60E-05 | **5.89E-03** |
| **FER** | 5q21.3 | 0.631 | 3.64E-05 | **5.90E-03** |
| **PALLD** | 4q32.3 | 0.631 | 3.71E-05 | **5.96E-03** |
| **PDGFB** | 22q13.1 | 0.629 | 3.93E-05 | **6.26E-03** |
| **ROR2** | 9q22.31 | 0.628 | 4.08E-05 | **6.40E-03** |
| **SDAD1** | 4q21.1 | 0.628 | 4.08E-05 | **6.40E-03** |
| **SMAD7** | 18q21.1 | 0.628 | 4.12E-05 | **6.40E-03** |
| **CAMSAP2** | 1q32.1 | 0.627 | 4.36E-05 | **6.70E-03** |
| **HIVEP1** | 6p24.1 | 0.626 | 4.44E-05 | **6.70E-03** |
| **SEC31A** | 4q21.22 | 0.626 | 4.48E-05 | **6.70E-03** |
| **STMN2** | 8q21.13 | 0.626 | 4.48E-05 | **6.70E-03** |
| **FBN1** | 15q21.1 | 0.625 | 4.52E-05 | **6.71E-03** |
| **LRRC32** | 11q13.5 | 0.625 | 4.57E-05 | **6.73E-03** |
| **PDZD2** | 5p13.3 | 0.625 | 4.61E-05 | **6.74E-03** |
| **MGP** | 12p12.3 | 0.624 | 4.74E-05 | **6.88E-03** |
| **SKIL** | 3q26.2 | 0.624 | 4.83E-05 | **6.95E-03** |
| **GPR176** | 15q14-q15.1 | 0.623 | 4.87E-05 | **6.97E-03** |
| **RUNX1T1** | 8q21.3 | 0.622 | 5.10E-05 | **7.23E-03** |
| **FGF1** | 5q31.3 | 0.621 | 5.25E-05 | **7.37E-03** |
| **IGF1R** | 15q26.3 | 0.621 | 5.30E-05 | **7.37E-03** |
| **POSTN** | 13q13.3 | 0.621 | 5.34E-05 | **7.37E-03** |
| **XIRP1** | 3p22.2 | 0.621 | 5.34E-05 | **7.37E-03** |
| **CCDC184** | 12q13.11 | 0.62 | 5.44E-05 | **7.42E-03** |
| **DACT1** | 14q23.1 | 0.62 | 5.49E-05 | **7.42E-03** |
| **MAML2** | 11q21 | 0.62 | 5.49E-05 | **7.42E-03** |
| **GAS7** | 17p13.1 | 0.619 | 5.80E-05 | **7.78E-03** |
| **DPYSL3** | 5q32 | 0.618 | 6.02E-05 | **8.02E-03** |
| **SYDE1** | 19p13.12 | 0.616 | 6.24E-05 | **8.26E-03** |
| **BMPR2** | 2q33.1-q33.2 | 0.616 | 6.30E-05 | **8.28E-03** |
| **AMPH** | 7p14.1 | 0.616 | 6.36E-05 | **8.30E-03** |
| **OMD** | 9q22.31 | 0.615 | 6.47E-05 | **8.34E-03** |
| **EPHA3** | 3p11.1 | 0.615 | 6.47E-05 | **8.34E-03** |
| **PTGIS** | 20q13.13 | 0.615 | 6.65E-05 | **8.45E-03** |
| **RNF169** | 11q13.4 | 0.615 | 6.65E-05 | **8.45E-03** |
| **LMCD1** | 3p25.3 | 0.614 | 6.77E-05 | **8.55E-03** |
| **LOXL4** | 10q24.2 | 0.613 | 6.96E-05 | **8.70E-03** |
| **PPFIA2** | 12q21.31 | 0.613 | 6.98E-05 | **8.70E-03** |
| **PTAFR** | 1p35.3 | 0.613 | 7.08E-05 | **8.77E-03** |
| **CLIC4** | 1p36.11 | 0.613 | 7.15E-05 | **8.80E-03** |
| **GREM1** | 15q13.3 | 0.612 | 7.21E-05 | **8.82E-03** |
| **NCK1** | 3q22.3 | 0.612 | 7.34E-05 | **8.92E-03** |
| **COLEC12** | 18p11.32 | 0.611 | 7.54E-05 | **9.11E-03** |
| **GALNT17** | 7q11.22 | 0.61 | 7.95E-05 | **9.40E-03** |
| **HAND2-AS1** | 4q34.1 | 0.61 | 7.95E-05 | **9.40E-03** |
| **SLITRK4** | Xq27.3 | 0.609 | 7.98E-05 | **9.40E-03** |
| **HOPX** | 4q12 | 0.608 | 8.24E-05 | **9.65E-03** |
| **FKBP7** | 2q31.2 | 0.608 | 8.38E-05 | **9.71E-03** |
| **SPARC** | 5q33.1 | 0.608 | 8.38E-05 | **9.71E-03** |
| **FMOD** | 1q32.1 | 0.607 | 8.53E-05 | **9.76E-03** |
| **DAAM1** | 14q23.1 | 0.607 | 8.53E-05 | **9.76E-03** |
| **GRP** | 18q21.32 | 0.607 | 8.81E-05 | **0.01** |
| **FAM135B** | 8q24.23 | 0.606 | 9.03E-05 | **0.0102** |
| **RAB31** | 18p11.22 | 0.605 | 9.15E-05 | **0.0102** |
| **CYP7B1** | 8q12.3 | 0.605 | 9.15E-05 | **0.0102** |
| **NR2F1** | 5q15 | 0.605 | 9.15E-05 | **0.0102** |
| **ST5** | 11p15.4 | 0.605 | 9.31E-05 | **0.0103** |
| **VCL** | 10q22.2 | 0.605 | 9.40E-05 | **0.0103** |
| **CPXM2** | 10q26.13 | 0.604 | 9.65E-05 | **0.0105** |
| **DYNC2H1** | 11q22.3 | 0.604 | 9.65E-05 | **0.0105** |
| **CCDC80** | 3q13.2 | 0.603 | 9.99E-05 | **0.0108** |
| **SORCS2** | 4p16.1 | 0.603 | 1.01E-04 | **0.0108** |
| **VGLL3** | 3p12.1 | 0.602 | 1.03E-04 | **0.0109** |
| **SEPTIN8** | 5q31.1 | 0.602 | 1.04E-04 | **0.0111** |
| **NRK** | Xq22.3 | 0.599 | 1.13E-04 | **0.0118** |
| **MTMR2** | 11q21 | 0.599 | 1.14E-04 | **0.0119** |
| **LRRC15** | 3q29 | 0.599 | 1.15E-04 | **0.0119** |
| **TIMP3** | 22q12.3 | 0.598 | 1.16E-04 | **0.0119** |
| **LINC00312** | 3p25.3 | 0.598 | 1.16E-04 | **0.0119** |
| **GLP2R** | 17p13.1 | 0.598 | 1.18E-04 | **0.012** |
| **FAM180A** | 7q33 | 0.598 | 1.19E-04 | **0.0121** |
| **GAP43** | 3q13.31 | 0.597 | 1.23E-04 | **0.0124** |
| **CHSY3** | 5q23.3 | 0.596 | 1.24E-04 | **0.0124** |
| **SLC6A14** | Xq23 | 0.596 | 1.25E-04 | **0.0124** |
| **SLC28A3** | 9q21.32-q21.33 | 0.596 | 1.25E-04 | **0.0124** |
| **KLHL30** | 2q37.3 | 0.596 | 1.26E-04 | **0.0124** |
| **THBS1** | 15q14 | 0.596 | 1.26E-04 | **0.0124** |
| **NEK1** | 4q33 | 0.595 | 1.28E-04 | **0.0126** |
| **SLC9A7** | Xp11.3\|Xp11.3 | 0.593 | 1.39E-04 | **0.0136** |
| **LAMC2** | 1q25.3 | 0.592 | 1.42E-04 | **0.0138** |
| **PDE1A** | 2q32.1 | 0.592 | 1.43E-04 | **0.0138** |
| **MIR99AHG** | 21q21.1 | 0.592 | 1.44E-04 | **0.0139** |
| **ANKRD50** | 4q28.1 | 0.592 | 1.45E-04 | **0.0139** |
| **EEF1DP3** | 13q13.1 | 0.59 | 1.51E-04 | **0.0144** |
| **TIMP2** | 17q25.3 | 0.59 | 1.51E-04 | **0.0144** |
| **MMP2** | 16q12.2 | 0.59 | 1.54E-04 | **0.0145** |

**Supplementary Table 2. The top 200 genes negatively correlated with COMP.**

| **Correlated Gene** | **Cytoband** | **Spearman's Correlation** | **p-Value** | **q-Value** |
| --- | --- | --- | --- | --- |
| **ATP5IF1** | 1p35.3 | -0.711 | 1.15E-06 | **4.66E-04** |
| **HSD17B3** | 9q22.32 | -0.692 | 2.89E-06 | **9.36E-04** |
| **LYPLA2** | 1p36.11 | -0.644 | 2.26E-05 | **4.39E-03** |
| **SLC22A3** | 6q25.3 | -0.626 | 4.48E-05 | **6.70E-03** |
| **MXD3** | 5q35.3 | -0.61 | 7.95E-05 | **9.40E-03** |
| **ZNF513** | 2p23.3 | -0.6 | 1.11E-04 | **0.0117** |
| **PPIEL** | 1p34.3 | -0.588 | 1.63E-04 | **0.015** |
| **COX4I1** | 16q24.1 | -0.582 | 1.95E-04 | **0.0168** |
| **DUS1L** | 17q25.3 | -0.58 | 2.08E-04 | **0.0174** |
| **ANKRD54** | 22q13.1 | -0.579 | 2.18E-04 | **0.0178** |
| **TMEM86B** | 19q13.42 | -0.576 | 2.38E-04 | **0.0191** |
| **TMEM160** | 19q13.32 | -0.575 | 2.46E-04 | **0.0195** |
| **GLYCTK** | 3p21.2 | -0.569 | 2.95E-04 | **0.0222** |
| **COMMD4** | 15q24.2 | -0.568 | 2.99E-04 | **0.0224** |
| **CPTP** | 1p36.33 | -0.565 | 3.33E-04 | **0.0237** |
| **BLOC1S1** | 12q13.2 | -0.56 | 3.82E-04 | **0.0263** |
| **AURKAIP1** | 1p36.33 | -0.56 | 3.85E-04 | **0.0264** |
| **NUDC** | 1p36.11 | -0.558 | 4.03E-04 | **0.0272** |
| **ULK3** | 15q24.1 | -0.558 | 4.06E-04 | **0.0272** |
| **FASTK** | 7q36.1 | -0.558 | 4.12E-04 | **0.0273** |
| **HSD17B10** | Xp11.22 | -0.557 | 4.15E-04 | **0.0274** |
| **SLC66A1** | 1p36.13 | -0.553 | 4.71E-04 | **0.0293** |
| **APOM** | 6p21.33 | -0.552 | 4.81E-04 | **0.0295** |
| **SFXN5** | 2p13.2 | -0.548 | 5.45E-04 | **0.0318** |
| **TRMU** | 22q13.31 | -0.546 | 5.69E-04 | **0.0322** |
| **GINS3** | 16q21 | -0.546 | 5.77E-04 | **0.0324** |
| **TXN2** | 22q12.3 | -0.545 | 5.90E-04 | **0.033** |
| **ZCCHC17** | 1p35.2 | -0.539 | 6.95E-04 | **0.0357** |
| **HMGN2** | 1p36.11 | -0.538 | 7.04E-04 | **0.0358** |
| **GSTK1** | 7q34 | -0.538 | 7.04E-04 | **0.0358** |
| **HMGCL** | 1p36.11 | -0.538 | 7.14E-04 | **0.0358** |
| **DIABLO** | 12q24.31 | -0.537 | 7.30E-04 | **0.036** |
| **MRPS15** | 1p34.3 | -0.537 | 7.40E-04 | **0.0361** |
| **ABCB8** | 7q36.1 | -0.537 | 7.40E-04 | **0.0361** |
| **ACAD10** | 12q24.12 | -0.534 | 8.04E-04 | **0.0379** |
| **PEF1** | 1p35.2 | -0.532 | 8.39E-04 | **0.0393** |
| **PDZD9** | 16p12.2 | -0.532 | 8.48E-04 | **0.0395** |
| **FAM136A** | 2p13.3 | -0.53 | 8.86E-04 | **0.0404** |
| **CRIP3** | 6p21.1 | -0.527 | 9.52E-04 | **0.0423** |
| **SELENOO** | 22q13.33 | -0.526 | 9.88E-04 | **0.0434** |
| **FITM1** | 14q12 | -0.526 | 9.95E-04 | **0.0434** |
| **RPS6KB2** | 11q13.2 | -0.524 | 1.04E-03 | **0.0444** |
| **TF** | 3q22.1 | -0.523 | 1.07E-03 | **0.0455** |
| **MRPL38** | 17q25.1 | -0.522 | 1.09E-03 | **0.0458** |
| **MTERF2** | 12q23.3 | -0.522 | 1.09E-03 | **0.0458** |
| **PDXP** | 22q13.1 | -0.522 | 1.10E-03 | **0.0458** |
| **DDTL** | 22q11.23 | -0.521 | 1.12E-03 | **0.0459** |
| **ATP8B5P** | 9p13.3 | -0.521 | 1.12E-03 | **0.046** |
| **GTF3C6** | 6q21 | -0.521 | 1.12E-03 | **0.046** |
| **ORMDL3** | 17q21.1 | -0.519 | 1.19E-03 | **0.0471** |
| **SLC25A5-AS1** | Xq24 | -0.518 | 1.22E-03 | **0.0476** |
| **POLE** | 12q24.33 | -0.517 | 1.25E-03 | **0.0488** |
| **NDUFV1** | 11q13.2 | -0.516 | 1.27E-03 | **0.049** |
| **NCAPH2** | 22q13.33 | -0.516 | 1.27E-03 | **0.049** |
| **C5ORF60** | 5q35.3 | -0.515 | 1.30E-03 | **0.0496** |
| **LINC01512** | 6p21.1 | -0.514 | 1.33E-03 | 0.0503 |
| **BRICD5** | 16p13.3 | -0.514 | 1.34E-03 | 0.0505 |
| **COX6A1** | 12q24.31\|12q24.2 | -0.514 | 1.35E-03 | 0.0505 |
| **GPS1** | 17q25.3 | -0.513 | 1.38E-03 | 0.0506 |
| **SMIM12** | 1p34.3 | -0.513 | 1.39E-03 | 0.0508 |
| **DMAP1** | 1p34.1 | -0.511 | 1.44E-03 | 0.0519 |
| **NAPA** | 19q13.32-q13.33 | -0.51 | 1.49E-03 | 0.0525 |
| **GALM** | 2p22.1 | -0.508 | 1.56E-03 | 0.0545 |
| **PRODH2** | 19q13.12 | -0.508 | 1.57E-03 | 0.0546 |
| **TXNL4A** | 18q23 | -0.507 | 1.59E-03 | 0.0547 |
| **DDT** | 22q11.23 | -0.506 | 1.63E-03 | 0.0555 |
| **RTCA** | 1p21.2 | -0.505 | 1.67E-03 | 0.0562 |
| **ENOSF1** | 18p11.32 | -0.505 | 1.68E-03 | 0.0564 |
| **RPP25** | 15q24.2 | -0.504 | 1.71E-03 | 0.0569 |
| **SNRPA1** | 15q26.3 | -0.504 | 1.71E-03 | 0.0569 |
| **C16ORF70** | 16q22.1 | -0.504 | 1.74E-03 | 0.0573 |
| **LSM4** | 19p13.11 | -0.502 | 1.81E-03 | 0.0585 |
| **AKR7A3** | 1p36.13 | -0.502 | 1.83E-03 | 0.0586 |
| **GLTPD2** | 17p13.2 | -0.501 | 1.87E-03 | 0.0593 |
| **UQCR10** | 22q12.2 | -0.5 | 1.90E-03 | 0.0596 |
| **DIO1** | 1p32.3 | -0.5 | 1.90E-03 | 0.0596 |
| **SCRN2** | 17q21.32 | -0.5 | 1.91E-03 | 0.0596 |
| **RAD9A** | 11q13.2 | -0.499 | 1.96E-03 | 0.0608 |
| **PKLR** | 1q22 | -0.498 | 1.99E-03 | 0.061 |
| **UBE2J2** | 1p36.33 | -0.498 | 1.99E-03 | 0.061 |
| **EPN1** | 19q13.42 | -0.498 | 2.01E-03 | 0.0613 |
| **C9ORF43** | 9q32 | -0.497 | 2.03E-03 | 0.0619 |
| **NDUFS6** | 5p15.33 | -0.497 | 2.07E-03 | 0.0629 |
| **FABP1** | 2p11.2 | -0.496 | 2.11E-03 | 0.0636 |
| **VRK3** | 19q13.33 | -0.494 | 2.19E-03 | 0.0655 |
| **POP5** | 12q24.31 | -0.494 | 2.20E-03 | 0.0657 |
| **MAN2C1** | 15q24.2 | -0.493 | 2.26E-03 | 0.0667 |
| **ZNRF2** | 7p14.3 | -0.493 | 2.27E-03 | 0.0668 |
| **SAPCD1** | 6p21.33 | -0.492 | 2.28E-03 | 0.0669 |
| **KIF22** | 16p11.2 | -0.492 | 2.28E-03 | 0.0669 |
| **DNAJC8** | 1p35.3 | -0.492 | 2.28E-03 | 0.0669 |
| **ASCC2** | 22q12.2 | -0.492 | 2.33E-03 | 0.0675 |
| **CDK5RAP3** | 17q21.32 | -0.491 | 2.35E-03 | 0.068 |
| **OXER1** | 2p21 | -0.491 | 2.38E-03 | 0.0684 |
| **IFT27** | 22q12.3 | -0.491 | 2.38E-03 | 0.0684 |
| **CIAO2B** | 16q22.1 | -0.49 | 2.43E-03 | 0.0689 |
| **SLC66A2** | 18q23 | -0.49 | 2.44E-03 | 0.0691 |
| **GPATCH3** | 1p36.11 | -0.489 | 2.46E-03 | 0.0693 |
| **COPE** | 19p13.11 | -0.489 | 2.47E-03 | 0.0694 |
| **COA3** | 17q21.2 | -0.489 | 2.47E-03 | 0.0694 |
| **AURKC** | 19q13.43 | -0.488 | 2.55E-03 | 0.0708 |
| **AGMAT** | 1p36.21 | -0.488 | 2.55E-03 | 0.0708 |
| **BIN1** | 2q14.3 | -0.487 | 2.58E-03 | 0.0711 |
| **ZBTB8OS** | 1p35.1 | -0.487 | 2.59E-03 | 0.0712 |
| **TUFM** | 16p11.2 | -0.485 | 2.69E-03 | 0.0729 |
| **MYH3** | 17p13.1 | -0.485 | 2.74E-03 | 0.074 |
| **RCC1L** | 7q11.23 | -0.484 | 2.75E-03 | 0.0741 |
| **FAH** | 15q25.1 | -0.484 | 2.80E-03 | 0.0749 |
| **ACBD4** | 17q21.31 | -0.483 | 2.84E-03 | 0.0753 |
| **FMC1** | 7q34 | -0.483 | 2.84E-03 | 0.0753 |
| **EIF3I** | 1p35.2 | -0.483 | 2.86E-03 | 0.0753 |
| **PSMC5** | 17q23.3 | -0.483 | 2.87E-03 | 0.0756 |
| **CENPX** | 17q25.3 | -0.482 | 2.89E-03 | 0.0756 |
| **LINC00526** | 18p11.31 | -0.482 | 2.89E-03 | 0.0756 |
| **ATP5MPL** | 14q32.33 | -0.482 | 2.91E-03 | 0.0757 |
| **ASPSCR1** | 17q25.3 | -0.481 | 2.98E-03 | 0.0762 |
| **SULT2A1** | 19q13.33 | -0.481 | 2.99E-03 | 0.0765 |
| **ATF5** | 19q13.33 | -0.48 | 3.03E-03 | 0.0772 |
| **TCTA** | 3p21.31 | -0.48 | 3.03E-03 | 0.0772 |
| **AKR7A2** | 1p36.13 | -0.48 | 3.07E-03 | 0.0778 |
| **GSTZ1** | 14q24.3 | -0.48 | 3.08E-03 | 0.0781 |
| **HAGH** | 16p13.3 | -0.478 | 3.21E-03 | 0.0802 |
| **SHBG** | 17p13.1 | -0.477 | 3.24E-03 | 0.0806 |
| **UQCC2** | 6p21.31 | -0.476 | 3.31E-03 | 0.0817 |
| **LHPP** | 10q26.13 | -0.476 | 3.35E-03 | 0.082 |
| **CMC2** | 16q23.2 | -0.476 | 3.35E-03 | 0.082 |
| **MRPS11** | 15q25.3 | -0.476 | 3.37E-03 | 0.082 |
| **SERF2** | 15q15.3 | -0.476 | 3.37E-03 | 0.082 |
| **EPHX2** | 8p21.2-p21.1 | -0.476 | 3.37E-03 | 0.082 |
| **SLC22A10** | 11q12.3 | -0.475 | 3.42E-03 | 0.0828 |
| **CHTF18** | 16p13.3 | -0.475 | 3.45E-03 | 0.0833 |
| **ATP6V0B** | 1p34.1 | -0.473 | 3.61E-03 | 0.0859 |
| **TMEM219** | 16p11.2 | -0.472 | 3.63E-03 | 0.0861 |
| **TAC3** | 12q13.3 | -0.472 | 3.64E-03 | 0.0863 |
| **PSMB2** | 1p34.3 | -0.471 | 3.72E-03 | 0.0869 |
| **MLF2** | 12p13.31 | -0.471 | 3.72E-03 | 0.0869 |
| **ACOT8** | 20q13.12 | -0.471 | 3.78E-03 | 0.0878 |
| **CHCHD10** | 22q11.23 | -0.47 | 3.80E-03 | 0.0881 |
| **SSU72** | 1p36.33 | -0.47 | 3.82E-03 | 0.0882 |
| **GPD1** | 12q13.12 | -0.47 | 3.82E-03 | 0.0882 |
| **SDF2L1** | 22q11.21 | -0.47 | 3.85E-03 | 0.0885 |
| **AKR7L** | 1p36.13\|1p35-p36.1 | -0.469 | 3.91E-03 | 0.0897 |
| **ITGB3BP** | 1p31.3 | -0.468 | 4.02E-03 | 0.091 |
| **NDUFB1** | 14q32.12 | -0.467 | 4.05E-03 | 0.0913 |
| **ZNHIT1** | 7q22.1 | -0.467 | 4.05E-03 | 0.0913 |
| **HNF1A** | 12q24.31 | -0.466 | 4.14E-03 | 0.0923 |
| **SNX22** | 15q22.31 | -0.466 | 4.21E-03 | 0.0931 |
| **LIN7B** | 19q13.33 | -0.464 | 4.36E-03 | 0.0949 |
| **PXMP2** | 12q24.33 | -0.464 | 4.36E-03 | 0.0949 |
| **CHCHD2** | 7p11.2 | -0.463 | 4.43E-03 | 0.0959 |
| **MYL12B** | 18p11.31 | -0.463 | 4.43E-03 | 0.0959 |
| **IDH3B** | 20p13 | -0.463 | 4.48E-03 | 0.0962 |
| **PYY2** | 17q11.2 | -0.462 | 4.58E-03 | 0.0976 |
| **STX10** | 19p13.13 | -0.462 | 4.61E-03 | 0.0977 |
| **KRT85** | 12q13.13 | -0.461 | 4.70E-03 | 0.0991 |
| **TMEM39B** | 1p35.2 | -0.46 | 4.74E-03 | 0.0993 |
| **TMUB1** | 7q36.1 | -0.46 | 4.79E-03 | 0.0997 |
| **LSM10** | 1p34.3 | -0.46 | 4.79E-03 | 0.0997 |
| **FAM192A** | 16q13 | -0.459 | 4.87E-03 | 0.1 |
| **PHLDB3** | 19q13.31 | -0.459 | 4.87E-03 | 0.1 |
| **NOL12** | 22q13.1 | -0.459 | 4.87E-03 | 0.1 |
| **MST1** | 3p21.31 | -0.458 | 4.93E-03 | 0.1 |
| **NDUFAF8** | 17q25.3 | -0.458 | 4.95E-03 | 0.101 |
| **ACYP1** | 14q24.3 | -0.458 | 4.98E-03 | 0.101 |
| **LINC01816** | 2p13.3 | -0.458 | 4.98E-03 | 0.101 |
| **FAM166A** | 9q34.3 | -0.458 | 4.99E-03 | 0.101 |
| **ERI3** | 1p34.1 | -0.458 | 5.01E-03 | 0.101 |
| **C7ORF50** | 7p22.3 | -0.457 | 5.06E-03 | 0.101 |
| **LYPLA2P1** | 6p21.32 | -0.456 | 5.15E-03 | 0.102 |
| **CENPH** | 5q13.2 | -0.455 | 5.29E-03 | 0.104 |
| **TACO1** | 17q23.3 | -0.455 | 5.35E-03 | 0.104 |
| **UBAP1L** | 15q22.31 | -0.454 | 5.38E-03 | 0.104 |
| **RNF181** | 2p11.2 | -0.454 | 5.41E-03 | 0.104 |
| **COX5B** | 2q11.2 | -0.454 | 5.47E-03 | 0.105 |
| **UNC119** | 17q11.2 | -0.453 | 5.56E-03 | 0.106 |
| **AGXT2** | 5p13.2 | -0.452 | 5.64E-03 | 0.107 |
| **NDUFA1** | Xq24 | -0.452 | 5.65E-03 | 0.107 |
| **PGP** | 16p13.3 | -0.452 | 5.68E-03 | 0.107 |
| **CYP2D6** | 22q13.2 | -0.451 | 5.71E-03 | 0.107 |
| **NFS1** | 20q11.22 | -0.451 | 5.77E-03 | 0.107 |
| **DHRS4** | 14q11.2 | -0.451 | 5.77E-03 | 0.107 |
| **COX6B1** | 19q13.12 | -0.451 | 5.81E-03 | 0.108 |
| **SDHB** | 1p36.13 | -0.45 | 5.87E-03 | 0.108 |
| **ZYG11A** | 1p32.3 | -0.45 | 5.88E-03 | 0.108 |
| **ATAD3B** | 1p36.33 | -0.45 | 5.93E-03 | 0.108 |
| **FUCA2** | 6q24.2 | -0.45 | 5.93E-03 | 0.108 |
| **ATP6V1E2** | 2p21\|2p16-p12 | -0.45 | 5.93E-03 | 0.108 |
| **GRAMD4** | 22q13.31 | -0.45 | 5.93E-03 | 0.108 |
| **SNRNP25** | 16p13.3 | -0.449 | 6.03E-03 | 0.109 |
| **OXLD1** | 17q25.3 | -0.449 | 6.03E-03 | 0.109 |
| **RBCK1** | 20p13 | -0.449 | 6.03E-03 | 0.109 |
| **FAHD2A** | 2q11.1 | -0.449 | 6.03E-03 | 0.109 |
| **GPRC5C** | 17q25.1 | -0.449 | 6.06E-03 | 0.109 |
| **IDH3G** | Xq28 | -0.449 | 6.06E-03 | 0.109 |
| **ACO2** | 22q13.2 | -0.448 | 6.09E-03 | 0.109 |
| **MEAF6** | 1p34.3 | -0.448 | 6.13E-03 | 0.109 |
| **DPY30** | 2p22.3 | -0.448 | 6.19E-03 | 0.11 |
| **XKR8** | 1p35.3 | -0.448 | 6.19E-03 | 0.11 |
| **RNF167** | 17p13.2 | -0.448 | 6.19E-03 | 0.11 |
| **SLC39A2** | 14q11.2 | -0.448 | 6.21E-03 | 0.11 |
